# Identification of an NDM-5-producing *Escherichia coli* Sequence Type 167 in a Neonatal Patient in China

**DOI:** 10.1038/srep29934

**Published:** 2016-07-13

**Authors:** Yuan-qi Zhu, Jing-yi Zhao, Cha Xu, Hui Zhao, Nan Jia, Yan-nian Li

**Affiliations:** 1 The Affiliated Hospital of Qingdao University, 16 Jiangsu Road, Qingdao 266003, China; 2 Center for Bioengineering and Biotechnology, China University of Petroleum (East China), 66 Changjiang West Road, Qingdao 266580, China; 3 Medical College, Qingdao University, 38 Dengzhou Road, Qingdao 266021, China

## Abstract

Emergence of New Delhi metallo-β-lactamase-producing *Enterobacteriaceae* has become a challenging threat to public health. Two carbapenem-resistant *Escherichia coli*, strain QD28 and QD29, were recovered from the aspirating sputum of a neonate and the urine of an adult in a Chinese hospital in 2013. Molecular typing revealed that both isolates belonged to the sequence type 167, but they were clonally diverse. Both isolates exhibited resistance to carbapenems, cephalosporins, ciprofloxacin, gentamicin, piperacillin-tazobactam and trimethoprim-sulfamethoxazole. In addition, strain QD28 was also resistant to aztreonam, and strain QD29 was resistant to amikacin, fosfomycin and minocycline. Antimicrobial resistance gene screening revealed that strain QD28 harbored *aac*(*6*′)*-Ib, bla*_CTX-M-14_, *bla*_NDM-5_, *bla*_TEM-1_ and *sul1* genes, and strain QD29 harbored *aac*(*6*′)*-Ib, bla*_CTX-M-3_, *bla*_NDM-5_, *bla*_TEM-1_, *rmtB, sul1* and *sul2* genes. The *bla*_NDM-5_ gene was found to be located on a 46-kb plasmid in two isolates, and further sequence analysis showed that this plasmid was highly similar to the previously reported IncX3 plasmid pNDM-MGR194 in India. This is the first identification of *bla*_NDM-5_-carrying *E. coli* in the neonatal infection.

Carbapenems have been traditionally recognized as a last resort treatment for severe infected diseases caused by multidrug-resistant gram-negative bacilli^1^. However, due to their increasing usage, a variety of carbapenem-resistant bacteria have emerged and posed a serious public health threat. These bacteria can produce different carbapenemases to inactivate the drugs, of which New Delhi metallo-β-lactamase (NDM) is the recent emerging one of clinical significance. NDM-1 was first identified from a *Klebsiella pneumoniae* isolate in India in 2008^2^, and has rapidly spread throughout the world. It can efficiently hydrolyze all β-lactams except monobactams. So far, 16 *bla*_NDM_ gene variants have been discovered and assigned in the Lahey Clinic database, and some variants are found to confer elevated carbapenem resistance^3^,^4^,^5^. The rapid evolution and dissemination of NDMs represent a crucial challenge for clinical infection treatments.

NDM-5 was discovered in a multidrug-resistant *Escherichia coli* isolate in the United Kingdom (UK) in 2011^3^. It has two amino acid substitutions (Val88Leu and Met154Leu) in comparison with NDM-1, and seems to confer increased resistance to extended-spectrum cephalosporins and carbapenems. In the last five years, its encoding gene *bla*_NDM-5_ has been identified in clinical isolates in Algeria^6^, America^7^, Australia^8^, China^9^, Denmark^10^, India^5^,^11^, Japan^12^, Poland^13^, Singapore^14^, South Korea^15^, Spain^16^ and the Netherlands^17^. Different sequence types (STs) of *E. coli* and *K. pneumoniae* have been detected as NDM-5 producers. The diversity of these isolates indicates probable horizontal transfer of the *bla*_NDM-5_ gene either through plasmids or the transposon-related mobile elements. A number of plasmids belonging to the IncF, IncFII, IncN and IncX3 incompatibility groups are reported to carry *bla*_NDM-5_ (Table 1), and some plasmids have been completely sequenced. Unlike the prevalent *bla*_NDM-1_ gene, *bla*_NDM-5_ has been detected only in sporadic cases nowadays. However, considering its increased resistance phenotypes and global distribution, epidemiological survey of *bla*_NDM-5_ should arouse our attention.

In this study, we detected two NDM-5-producing *E. coli* isolates from the hospitalized patients in a Chinese hospital, of which one strain was isolated from a neonate and resistant to a range of antimicrobials.

## Results

### Strain features

**Figure 1:**
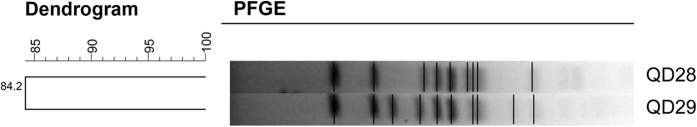
Dendrogram of patterns generated by pulsed-field gel electrophoresis (PFGE) of strain QD28 and QD29 using the BioNumerics software program .

Two carbapenem-resistant *E. coli* isolates were collected from two hospitalized patients in different wards in a hospital in Shandong Province, China during September to October in 2013. Both patients had no history of foreign travel. Positive results were obtained in the modified Hodge test (MHT) and metallo-β-lactamase (MBL) antimicrobial gradient test, suggesting that they can produce MBLs. Further carbapenemase gene screening revealed that both isolates carried the *bla*_NDM-5_ gene. Because NDM-5 producers are infrequently detected worldwide, we performed multilocus sequence typing (MLST) and pulsed field gel electrophoresis (PFGE) experiments to analyze their clonal relatedness. Both isolates belonged to the ST167 epidemic clone, but PFGE revealed that they can be classified into different pulsotypes (Fig. 1), indicating that they were clonally diverse and excluding the possibility of nosocomial cross-transmission.

Antimicrobial susceptibility testing also showed different resistance profiles of these two *E. coli* isolates (Table 2). Strain QD28 displayed resistance to aztreonam, carbapenems (ertapenem, imipenem and meropenem), cephalosporins (cefotaxime, ceftazidime, cefepime and cefoxitin), ciprofloxacin, gentamicin, piperacillin-tazobactam and trimethoprim-sulfamethoxazole, intermediate resistance to minocycline and tobramycin, and susceptibility to amikacin, fosfomycin, polymyxin B and tigecycline. However, strain QD29 was high level resistant to most tested antimicrobial compounds, and only susceptible to aztreonam, polymyxin B and tigecycline.

### Antimicrobial resistance gene screening

According to the multidrug resistance phenotypes of two isolates, a variety of antimicrobial resistance genes were screened by PCR in our study. In addition to *bla*_NDM-5_, strain QD28 was found to carry the *aac*(*6*′)*-Ib, bla*_CTX-M-14_, *bla*_TEM-1_ and *sul1* genes, and strain QD29 carried the *aac*(*6*′)-*Ib, bla*_CTX-M-3_, *bla*_TEM-1_, *rmtB, sul1* and *sul2* genes. The AmpC-type β-lactamase genes, plasmid-mediated fosfomycin resistance (PMFR) genes and plasmid-mediated quinolone resistance (PMQR) genes were not detected. Moreover, both isolates had amino acid substitutions in the quinolone resistance-determining regions (QRDRs), for strain QD28 in GyrA (Ser83Leu and Asp87Asn) and ParC (Ser80Ile), and for strain QD29 in GyrA (Ser83Leu and Asp87Asn), ParC (Ser80Ile) and ParE (Leu416Phe).

### *bla*_NDM-5_-carrying plasmid analysis

In order to identify the *bla*_NDM-5_-carrying plasmids in two *E. coli* isolates, we carried out gene transfer experiments. The electroporation experiments were successful, but conjugation experiments failed. The *E. coli* TOP10 electroparants of strain QD28 and QD29 exhibited identical drug susceptibility phenotypes (Table 2). They were resistant to carbapenems, cephalosporins and piperacillin-tazobactam, but susceptible to the other non-β-lactam agents, in comparison with their *E. coli* donors. In addition, the *E. coli* electroparants of both isolates were found to contain a single plasmid of the same size. Only *bla*_NDM-5_ was identified in this plasmid, and the other resistance genes present in strain QD28 and QD29 were not detected. Therefore, we presumed that the *bla*_NDM-5_-carrying plasmids in strain QD28 and QD29 might be very similar.

**Figure 2:**
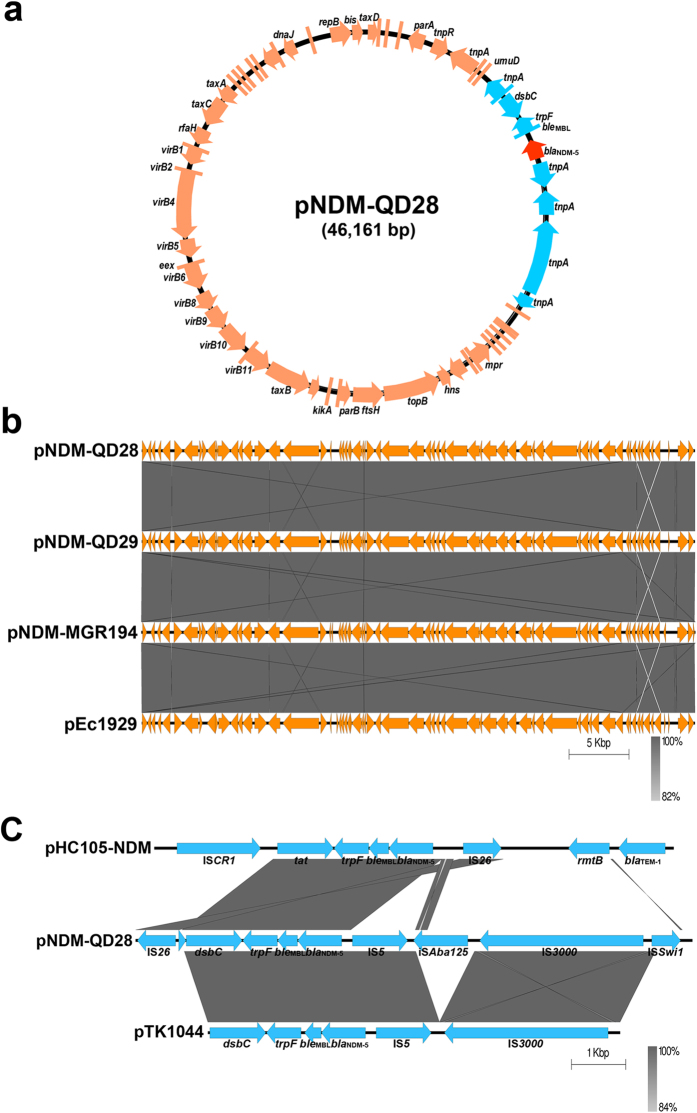
Plasmid analysis of pNDM-QD28. Schematic map of plasmid pNDM-QD28 (**a**), comparative analysis of four *bla*_NDM-5_-carrying IncX3 plasmids (**b**), and comparative analysis of the genetic contexts of *bla*_NDM-5_ in IncFII, IncX3 and IncN plasmids (**c**). The putative open reading frames are shown as arrowheads or rods (less than 130 amino acids). The gene name is shown near the corresponding arrowhead or rod. The depth of shading is indicative of the percentage BLASTN match, as indicated bottom.

Subsequently, we attempted to obtain the whole plasmid sequences to better characterize these two *bla*_NDM-5_-carrying plasmids. The nucleotide sequences of pNDM-QD28 were obtained using Miseq techniques, and the potential errors were corrected by PCR mapping. The pNDM-QD29 sequences were obtained by PCR mapping. Sequence analysis showed that these two plasmids with 46 kb in length were almost identical (Fig. 2), and had only four base substitutions. They belonged to the IncX3 group based on PlasmidFinder analysis on Center for Genomic Epidemiology. Further sequence alignments on BLAST revealed that the plasmid sequences showed more than 99% identities with those of the previously described plasmid pNDM-MGR194 of *K. pneumoniae* MGR-K194 in India^11^. The *bla*_NDM-5_ gene was preceded by IS*Swi1*, IS*3000*, IS*Aba125* and IS*5*, and followed by *ble*_MBL_ (encoding resistance to bleomycin), *trpF, dsbC* and IS*26*. Other antimicrobial resistance genes were not detected in this plasmid.

Comparative analysis of the genetic contexts of *bla*_NDM-5_ in this IncX3 plasmid and the previously reported IncN^12^ and IncFII^16^ plasmids revealed some differences (Fig. 2c). In plasmid pNDM-QD28, the IS*Aba125* was interrupted by the insertion of IS*5*, so an IS*Aba125* remnant was present between IS*5* and IS*3000*. However, this remnant was missing in plasmid pTK1044, suggesting that additional gene deletion and rearrangement may occur in this IncN plasmid. In plasmid pHC105-NDM, the *bla*_NDM-5_ module (*bla*_NDM-5_-*ble*_MBL_-*trpF*-*tat*) was surrounded by IS*26* and IS*CR1* that were associated with the *Δ*Tn3 transposon and class I integron^16^, respectively, indicating that *bla*_NDM-5_ in this IncFII plasmid was mediated by a distinct mobilization mechanism.

Plasmid stability experiments showed that plasmids pNDM-QD28 and pNDM-QD29 were stable in isolate QD28 and QD29. After 10 rounds of subculture in MacConkey agar without antibiotic addition, the randomly selected strains all contained a plasmid identical to pNDM-QD28 and pNDM-QD29 in size, and they all harbored the *bla*_NDM-5_ gene.

## Discussion

Emergence of NDM-producing *Enterobacteriaceae* has become a crucial issue of global concern. The *bla*_NDM_ gene not only confers resistance to most β-lactams, but also accompanies with multiple resistance gene determinants of different groups of antimicrobials in the same strain, which enables pathogens to become multidrug resistant. Therefore, NDM producers lead to limited clinical therapeutic options, and represent a significant threat to public health. Epidemiological investigation and surveillance of NDMs are of importance to clinical infection control. Our study reported two NDM-5-producing *E. coli* isolates from Chinese hospitalized patients in 2013.

Strain QD28 was collected from a newborn admitted with neonatal pneumonia, which should be of significant note. NDM-producing *Enterobacteriaceae* are rarely identified in neonatal infections, and only a few cases have been reported so far. In the last five years, some *bla*_NDM-1_-positive clinical isolates of *Citrobacter* sp., *E. coli, Enterobacter cloacae* and *K. pneumoniae* have been detected in neonates diagnosed with pneumonia, sepsis and septicaemia in India^18^,^19^,^20^,^21^, and NDM-1-producing *K. pneumoniae* isolates have been identified in neonatal units in Colombia^22^, Turkey^23^ and UK^24^. In addition, three NDM-1-producing *Acinetobacter baumannii* isolates were detected in association with neonatal infections in China^25^. To the best of our knowledge, our study is the first identification of NDM-5-producing *E. coli* in the neonatal infection. Other patients in the same ward were also screened for carbapenem-resistant isolates, but no NDM-5 producers were observed. The hospital environments were screened for multidrug resistant isolates each month, and no NDM-5 producers were observed. Therefore, the origin of isolate QD28 was unclear.

Aminoglycosides and fluoroquinolones are rarely used for neonates and infants in China. However, strain QD28 displayed resistance to gentamicin and ciprofloxacin. Emergence of the resistance gene *aac*(*6*′)*-Ib* and mutations in QRDRs can be responsible for the resistance to the above two antimicrobial compounds.

The neonates are immunocompromised patients with a high risk of infection. Identification of multidrug-resistant NDM producers in neonatal infections is extremely worrisome, which will be very difficult to treat. Therefore, infection control measures should be reinforced to reduce the hospital-acquired multidrug resistance in the near future, such as implementing strict contact isolation precautions and performing supervised disinfection procedures.

Strain QD29 was recovered from a 47-year-old woman who contacted more complicated environments than neonates. Therefore, it displayed more diverse resistance profiles in comparison with strain QD28. Except aztreonam, polymyxin B and tigecycline, strain QD29 can be resistant to the rest antimicrobials tested in our study. In addition, strain QD29 exhibited higher level resistance to aminoglycosides, which may be explained by carrying the *rmtB* gene.

MLST analysis revealed that both NDM-5-producing *E. coli* isolates QD28 and QD29 in our study belonged to ST167, which is an internationally disseminated pathogen of human and animal, and associated with numerous resistance mechanisms, especially *bla*_NDM_^26^,^27^. The *bla*_NDM-1_, *bla*_NDM-5_ and *bla*_NDM-7_ genes have been detected in *E. coli* ST167 as reported previously^7^,^9^,^28^,^29^. In particular, the NDM-5 producers hitherto identified in China are *E. coli* ST167 (8 isolates), ST2608 (1 isolate) and ST5131 (1 isolate)^9^,^30^,^31^, indicating that ST167 is an important reservoir of *bla*_NDM-5_ in China.

The *bla*_NDM-5_-carrying plasmids pNDM-QD28 and pNDM-QD29 were found to have almost identical nucleotide sequences with the previously reported IncX3 plasmid pNDM-MGR194 in *K. pneumoniae* in India^11^. In addition, the *bla*_NDM-5_-carrying plasmids, such as pEc1929 and pNDM5_0215, which were detected previously in five isolates from distinct cities in China, also showed high identities with pNDM-MGR194, except some minor differences^9^,^31^. Therefore, we presumed that this pNDM-MGR194-like plasmid probably played a significant role in the emergence and dissemination of the *bla*_NDM-5_ gene in China.

Furthermore, the *bla*_NDM-5_ genes identified in Australia and Denmark were also found to be located in IncX3 plasmids highly similar to pNDM-MGR194^8^,^10^. Their *E. coli* carriers (ST648 and ST1284) were isolated from patients who travelled to India (Table 1), suggesting that these two cases were associated with Indian NDM-5 dissemination. This pNDM-MGR194-like plasmid is consequently an important vehicle for the international dissemination of *bla*_NDM-5_. Identification of pNDM-MGR194-like plasmids in *K. pneumoniae* and *E. coli* of different STs indicates that this plasmid is able to mediate inter- and intra-species transfer of *bla*_NDM-5_, which will facilitate the rapid distribution of *bla*_NDM-5_ among enterobacterial species.

Although international travel and multinational medical treatment are reported to contribute greatly to the global distribution of *bla*_NDM_ genes, all patients carrying NDM-5 producers identified in China had no foreign travel^9^,^30^,^31^. Therefore, these NDM-5-producing isolates are presumed to be autochthonous clones that have acquired the *bla*_NDM-5_-positive plasmid. Recent surveys have identified the NDM-5-producing *E. coli* from mastitic milk samples in India and dog in Algeria^32^,^33^, which should be of note, because farm animals and pets are important sources of antimicrobial resistant bacteria^34^. Although no NDM-5 producers have been reported in the environmental samples or animals in China, some NDM-1-producing *Acinetobacter* spp. have been identified in Chinese hospital sewages and animal farms^35^,^36^. More epidemiological studies are necessary in the future to clarify the mechanisms of emergence, evolution and dissemination of NDM-5 in China.

## Methods

### Ethics statement

Informed consent was obtained from the patients involved in this study. For the neonatal patient, consent from his parent was obtained. This study methods were reviewed and approved by the Ethics Committee of the Affiliated Hospital of Qingdao University and were carried out in accordance with the approved guidelines.

### Bacterial strains

Two carbapenem-resistant gram-negative bacteria were collected from a teaching hospital in Shandong Province, east of China during 2013. Strain QD28 was isolated from the aspirating sputum of a seven-day-old newborn on October 2, 2013. He was a preterm baby, 1.6 kg in weight. Apgar scores of the baby were 9 at 1 minute, 9 at 5 minutes, and 10 at 10 minutes. This low birth weight newborn was born in this hospital, admitted for neonatal pneumonia infection, and treated with meropenem. Strain QD29 was recovered from the urine sample of a 47-year-old female patient on September 15, 2013. She was diagnosed with urinary tract infection, and received ciprofloxacin and piperacillin/tazobactam after hospital admission. Both isolates were considered to be infections. Bacterial identification by using Vitek-2 compact system (BioMérieux, France) revealed that they were both *E. coli*. The MHT and MBL antimicrobial gradient test were performed for phenotypic identification of these two isolates.

### Molecular typing

PFGE was carried out to analyze the clonal relatedness between two *E. coli* isolates. The genomic DNA was prepared in agarose blocks and digested with *Xba*I according to the protocol described by the CDC PulseNet program^37^. The *Xba*I-digested genomic DNA was subjected to PFGE using a CHEF-Mapper XA PFGE System (Bio-Rad, USA).

MLST was also carried out for molecular typing. Bacterial genomic DNA was extracted from the *E. coli* isolate. Seven housekeeping genes (*adk, fumC, gyrB, icd, mdh, purA* and *recA*) were amplified by PCR^38^, and the amplicons were submitted for DNA sequencing to analyze the ST.

### Antimicrobial susceptibility testing

Antimicrobial susceptibility testing was performed on Mueller-Hinton agar plates using Etest strips (AB-BioMérieux, Solna, Sweden). The antibiotics tested in the study were amikacin, aztreonam, cefepime, cefotaxime, cefoxitin, ceftazidime, ciprofloxacin, ertapenem, fosfomycin, gentamicin, imipenem, meropenem, minocycline, piperacillin-tazobactam, polymyxin B, tigecycline, tobramycin and trimethoprim-sulfamethoxazole. The results were interpreted according to the Clinical and Laboratory Standards Institute guidelines^39^, except tigecycline and polymyxin B, for which European Committee on Antimicrobial Susceptibility Testing breakpoints were used^40^. *E. coli* ATCC 25922 and *Pseudomonas aeruginosa* ATCC 27853 were used as the control strains.

### Antimicrobial resistance gene screening

A variety of antimicrobial resistance genes were screened by PCR, and the positive amplicons were determined by DNA sequencing. These resistance genes included the prevalent extended-spectrum β-lactamase genes (*bla*_CTX-M_, *bla*_OXA_, *bla*_SHV_ and *bla*_TEM_)^41^,^42^, AmpC β-lactamase genes (*bla*_ACC_, *bla*_ACT_, *bla*_BIL_, *bla*_CMY_, *bla*_DHA_, *bla*_FOX_, *bla*_LAT_, *bla*_MIR_ and *bla*_MOX_)^43^, carbapenemase genes (*bla*_AIM_, *bla*_BIC_, *bla*_DIM_, *bla*_GIM_, *bla*_IMP_, *bla*_KPC_, *bla*_NDM_, *bla*_OXA-48_, *bla*_SIM_, *bla*_SPM_ and *bla*_VIM_)^44^, 16S rRNA methylase genes (*armA, npmA, rmtA, rmtB, rmtC* and *rmtD*)^45^, PMFR genes (*fosA, fosA3* and *fosC*), PMQR genes [*aac*(*6*′)*-Ib-cr, qepA, qnrA, qnrB, qnrC, qnrD* and *qnrS*]^46^, and dihydrofolate reductase genes (*sul1, sul2* and *sul3*)^47^.

### Mutations in the QRDRs

The QRDRs of the *gyrA, gyrB, parC* and *parE* genes in the chromosome were amplified by PCR using the primers previously described^48^,^49^, and sequenced to analyze the potential mutations.

### *bla*_NDM_-carrying plasmid analysis

Electroporation and conjugation experiments were carried out to identify the *bla*_NDM_-carrying plasmids present in the two *E. coli* isolates. Plasmids were individually extracted from the *E. coli* isolates using a Qiagen Plasmid Midi Kit (Qiagen, Germany), and then electroporated to the competent *E. coli* TOP10 recipient cells. The *E. coli* electroporants were selected on Luria-Bertani agar plates containing 6 mg L^−1^ ceftazidime. Conjugation experiments were conducted by liquid mating with *E. coli* J53Azi^R^ as the recipient cells, and the transconjugants were selected on tryptic soy agar plates containing 8 mg L^−1^ ceftazidime and 100 mg L^−1^ sodium azide. The *E. coli* electroporants were confirmed as *bla*_NDM_-positive by PCR analysis. The *bla*_NDM_-carrying plasmid size was evaluated by the S1-PFGE method^50^.

### Plasmid sequencing

The *bla*_NDM_-carrying plasmids pNDM-QD28 (present in strain QD28) and pNDM-QD29 (present in strain QD29) were individually extracted from the *E. coli* electroporants using a Qiagen Plasmid Midi Kit for further DNA sequencing. The whole plasmid sequences of pNDM-QD28 were determined using an Illumina Miseq platform. A DNA library was prepared and subjected to paired end (2 × 300 base run) sequencing at the Sangon Biotech (Shanghai) Co. Ltd in China. The raw sequencing data were pre-processed using Prinseq-lite, and subsequently assembled *de novo* using Velvet. The Gapcloser and Gapfiller programs were used for closing gaps, and PrinSeS-G was used for correcting sequence errors. Subsequently, we used the assembled plasmid sequences of pNDM-QD28 as reference, acquired the plasmid sequences of pNDM-QD29 and re-corrected the plasmid sequences of pNDM-QD28 by PCR mapping. The plasmid sequences were annotated by RAST^51^, and the predicted open reading frames were further compared against the non-redundant protein database using BLAST.

### Plasmid stability

Strain QD28 and QD29 was individually streaked out in the MacConkey agar, incubated at 37 °C for 24 h, and then transferred to a fresh MacConkey agar. After repeated streaking for 10 days, 10 individual colonies were randomly selected and used to extract their plasmids. Subsequently, PCR was carried out to screen the *bla*_NDM-5_ genes.

### Nucleotide sequences

The complete nucleotide sequences of plasmid pNDM-QD28 and pNDM-QD29 were submitted to GenBank with the accession numbers KU167608 and KU167609.

## Additional Information

**Accession Code**: The GenBank accession numbers of the reference sequences are as follows: the IncX3 plasmid pNDM-MGR194, KF220657; the IncX3 plasmid pEc1929, KT824791; the IncFII plasmid pHC105-NDM, KM598665; the IncN plasmid pTK1044, LC000627.

**How to cite this article**: Zhu, Y.-q. *et al*. Identification of an NDM-5-producing *Escherichia coli* Sequence Type 167 in a Neonatal Patient in China. *Sci. Rep.* **6**, 29934; doi: 10.1038/srep29934 (2016).

## Figures and Tables

**Table 1:** Detailed information of the *bla*_NDM-5_-carrying plasmids reported.

Inc group	Transferability^a^	Size (kb)	Host strain	MLST	Country	Age	Sex	Sample	Travel history	Reference
IncF	—	>100	*E. coli*	ST648	UK	41 years	—	Perineum and throat	India	3
	T	>100	*E. coli*	—	India	28 years	F	Intra-operative pus	—	5
	T	>100	*E. coli*	—	India	52 years	M	Intra-operative pus	—	5
IncFII	C	110	*E. coli*	ST448	Poland	17 years	F	Stool	India	13
	C	90	*E. coli*	ST448	Spain	78 years	F	Urine	No	16
IncN	C	110	*E. coli*	ST540	Japan	—	—	Feces	Bangladesh	12
IncX3	T	46^b^	*K. pneumoniae*	—	India	—	—	Blood	—	11
	—	46^b^	*E. coli*	ST1284	Denmark	74 years	F	Groin	India	10
	—	46^b^	*E. coli*	ST648	Australia	55 years	F	Urine	India	8
	C	46^b^	*E. coli*	ST167	China	75 years	M	Rectum	No	9
	C	46^b^	*E. coli*	ST167	China	80s	F	Urine	No	31
	C	46^b^	*E. coli*	ST167	China	20s	F	Blood	No	31
	C	46^b^	*E. coli*	ST2608	China	20s	M	Surgical wound swab	No	31
	C	46^b^	*E. coli*	ST5131	China	30s	F	Vaginal secretions	No	31
	T	46^b^	*E. coli*	ST167	China	7 days	M	Aspirating sputum	No	This study
	T	46^b^	*E. coli*	ST167	China	47 years	F	Urine	No	This study
untypeable	C	48	*K. pneumoniae*	ST231	Singapore	5 years	F	Urine	Bangladesh	14

^a^C: plasmid can transfer to *E. coli* recipients by conjugation; T: plasmid can transfer to *E. coli* recipients by transformation or electroporation.

^b^These plasmids are identical or near-identical to plasmid pNDM-MGR194.

**Table 2:** Minimal inhibitory concentrations (MICs) of a variety of antimicrobials for *Escherichia coli* strain QD28, *E. coli* strain QD29 and their *E. coli* electroporants.

Antimicrobials	MIC (mg L^−1^)				
	QD28	QD28-T^a^	QD29	QD29-T^b^	TOP10
Aztreonam	24	0.64	2	0.64	0.047
Cefepime	≥256	16	32	12	0.047
Cefotaxime	≥256	≥256	≥256	≥256	0.047
Cefoxitin	≥256	≥256	≥256	≥256	2
Ceftazidime	≥256	≥256	≥256	≥32	0.019
Ertapenem	≥32	12	≥32	8	0.38
Imipenem	≥32	16	≥32	12	0.38
Meropenem	≥32	8	≥32	4	0.47
Piperacillin-tazobactam	≥256	≥256	≥256	≥256	1
Amikacin	1.5	1.5	≥256	1	1
Gentamicin	32	0.25	≥256	0.38	0.25
Tobramycin	10	0.25	≥256	0.25	0.25
Ciprofloxacin	6	≤0.02	≥32	≤0.02	0.002
Fosfomycin	2	2	≥1024	2	2
Minocycline	6	1	32	0.5	1
Tigecycline	0.125	0.5	0.25	0.19	0.25
Trimethoprim-sulfamethoxazole	≥32	0.47	≥32	0.32	0.032
Polymyxin B	0.38	0.38	0.5	0.25	0.25

^a^*E. coli* electroporant of strain QD28.

^b^*E. coli* electroporant of strain QD29.
